# Retraction and remodeling of rod spherules are early events following experimental retinal detachment: an ultrastructural study using serial sections

**Published:** 2009-01-09

**Authors:** Kenneth A. Linberg, Geoffrey P. Lewis, Steven K. Fisher

**Affiliations:** 1 Neuroscience Research Institute, University of California, Santa Barbara, CA; 2 Department of Molecular, Cellular, and Developmental Biology, University of California, Santa Barbara, CA

## Abstract

**Purpose:**

To describe changes induced by retinal detachment in the ultrastructure and organization of rod terminals and their connections with B-type horizontal cell (HC) axon terminals and rod bipolar cell (RB) dendrites.

**Methods:**

Sections from control, 3 day, 7 day, and 28 day detached feline retinas were prepared for confocal immunofluorescence, light microscopy, and electron microscopy (EM). In addition, 100 μm-thick vibratome sections were immunolabeled with markers for photoreceptor terminals, HCs, and RBs. More than 40 rod spherules were studied in 90 nm-thick serial sections by transmission EM to greater detail changes in their ultrastructure and innervation.

**Results:**

Following retinal detachment, many rod terminals retracted varying distances toward their respective cell bodies in the outer nuclear layer (ONL). In retinas detached for 1 to 4 weeks, an altered synaptic vesicle population and associated ribbons were found in all retracting terminals. Many rod somata in the distal ONL seemed to lack synaptic terminal structures altogether. In a retina detached for 1 week, EM showed that less than half of the retracted terminals remain in contact with RB dendrites. In contrast, almost every surviving spherule was contacted by neurite outgrowths from the axon terminals of the B-type HC. Although retracted spherules had several presynaptic structures similar to those in normal retina, numerous changes occurred in their overall synaptic architecture. The spherule’s invagination was shallower, contained fewer postsynaptic processes, and often had “opened,” allowing swollen HC processes apposing the synaptic ribbon to directly contact other processes of the outer plexiform layer (OPL) neuropil. Whereas in normal cat retina each HC “lobe” comes from a different axon terminal system, after detachment, the opposing lateral elements can stem from the same terminal. The innervating RB dendrites that branched off stout RB dendritic trunks that extended up into the ONL were thinner than normal, unbranched, often electron dense, and lacked organelles. When present, most merely lay adjacent to retracting spherules rather than enter any synaptic invagination that might still occur.

**Conclusions:**

Immunocytochemistry enabled RB and HC neurites to appear postsynaptic to retracted rod terminals. However, at the ultrastructural level, HCs seemed to more consistently retain connection with the retracted spherules than the RBs. The highly conserved architecture of the rod spherule was lost as the invagination opened and postsynaptic contacts became fewer. It would seem that the lack of RB central elements as well as the drastic alterations in the architecture of most retracted terminals would necessarily alter the physiology of this complex synapse.

## Introduction

Most central nervous system (CNS) neurons have multiple, often hundreds of presynaptic active zones. The mammalian rod photoreceptor is unique in its reliance on a single, complex presynaptic structure with a large active zone [1,2]. In feline retina where the rods number 400,000/mm^2^ over much of the retina [3], the postsynaptic targets of their terminals are the axon telodendria of the B-type horizontal cell (HC) and rod bipolar cell (RB) dendrites [4,5]. Several investigations have shown that most of the pre- and postsynaptic components of this spherule complex are essentially the same across mammalian species. For example, the height and width of the arcuate synaptic ribbon, the surface area of the active zone itself, and the volume of extracellular space within the deep invagination are virtually the same from cat to rat to human; moreover, unlike the output terminals of most retinal neurons, rod spherules change little in size with increasing distance from visual centers, such as a fovea or area centralis [1,6]. This highly conserved microstructure argues for a successful solution to the physiologic and energetic demands of a synapse where glutamate release is almost continuously varying with light intensity [7]. The cytoarchitecture of this ribbon synaptic complex provides a mechanism for the high rates of sustained transmitter release by vesicle exocytosis [1,2,8-11]. The structural organization of the spherule underlies the sensitivity of the rod photoreceptor and its ability to rapidly accommodate changes in illumination [12]. It would seem that changes in any component of this synaptic complex have the potential to alter the rod’s communication with second order neurons. Our earlier studies suggested that retinal detachment produces a widespread disorganization of rod terminals in the outer plexiform layer (OPL) with many retracting deep into the outer nuclear layer (ONL) [13,14]. These observations indicate that these synapses may be physically altered in ways that could prevent normal synaptic transmission.

There is a large body of existing literature on retinal circuitry in the feline retina [4,5,15-20]. Detailed information about the organization of its rod synapse [1,2,6] makes the cat retina a good choice for studying changes in rod connectivity as a result of detachment.

It has been shown previously that between 1 and 3 days after detachment, rod terminals begin retracting [13] and both HCs and RBs sprout neurites that grow into the ONL [14]. While the stout RB outgrowths tend to be single, short, and unbranching, the processes from B-type HC axon terminals can occur singly or in clusters and can achieve great lengths [14,21], often growing out of the neural retina into the subretinal space. A similar pattern of terminal retraction and neurite outgrowth from HCs and RBs has also been reported in the late stages of the RCS retinal degeneration [22], retinal degenerations associated with the *nob2* mutant mouse [23,24]; with the knockouts of bassoon [25,26], CaBP2 [27], and retinoschisin [28]; with the mid-stages of the double rod–cone knockout [29]; as well as in retinopathy induced by vigabatrin [30]—all suggesting that they are common responses to photoreceptor degeneration. Moreover, the retraction of photoreceptor axons and terminals has also been reported in cultured porcine retinal explants prepared for transplantation studies [31], a phenomenon that can be prevented by treating the explants with a cAMP analog or the adenylyl cyclase stimulant, forskolin [32]. Prior to this there has been no detailed high resolution studies of the transformations that rod synapses undergo as spherules pull back from the OPL or of the RB and HC axon terminal neurites (HCat) neurites that appear to grow toward them in what may be an attempt to maintain physiologic connection.

## Methods

Experimental retinal detachments were made in adult cat eyes (*Felis domesticus*) as previously described [33]. Briefly, a solution of sodium hyaluronate, consisting of 0.25% Healon in balanced salt solution (Pharmacia, Piscataway, NJ), was infused via a fine glass micropipette between the retina and the retinal pigment epithelium (RPE). The Healon is needed to maintain the detachment.

The experimental use of the animals was conducted in compliance with both the guidelines of the UCSB IACUC and the ARVO Statement for the Use of Animals in Ophthalmic and Vision Research. Cats were maintained on a 12:12 light dark cycle under ordinary room illumination. The specimens examined in this study stemmed from earlier investigations.

### Immunocytochemistry and confocal microscopy

The samples consisted of 100 μm agarose-embedded sections of normal cat retina and retinas that had been detached for 3, 7, or 28 days. Tissue was fixed and held in 4% paraformaldehyde in sodium cacodylate buffer until they were sectioned without dehydration as described previously [14,34]. Sections were blocked for 2 h to overnight at 4 °C in 1:20 normal donkey serum (Jackson ImmunoResearch Laboratories, West Grove, PA) in PBS containing 0.5% BSA, 0.1% Triton X-1009, and 0.1% sodium azide (PBTA). Sections were then incubated overnight in various combinations of the following primary antibodies all diluted with PBTA: 1:500 calretinin (rabbit polyclonal; Chemicon, Temecula, CA), 1:100 α-protein kinase C (rabbit polyclonal; BioMol Research Labs, Plymouth Meeting, PA), 1:100 biotinylated neurofilament (mouse monoclonal 70 and 200 kDa subunits; Biomeda, Foster City, CA; biotinylation done by Vector Labs, Burlingame, CA), and 1:500 VAMP2 (synaptobrevin, mouse monoclonal; Synaptic Systems GmbH Göttingen, Germany). These primary antibodies were visualized using secondary antibodies conjugated with various fluorochromes: donkey anti-rabbit or anti-mouse immunoglobulin G conjugated to either 1:200 Cy3 or Cy2 (Jackson ImmunoResearch Laboratories), and streptavidin conjugated to 1:100 Cy5 (Jackson ImmunoResearch Laboratories). Samples were rinsed, mounted in 5% n-propyl gallate in glycerol, and examined with a BioRad 1024 confocal microscope (BioRad, Hercules, CA). Optical slices of 0.5 μm were viewed with a 40X/1.3 N.A. objective. Some images consisted of multiple planes of focus projected as a z-series; the number of these planes is included in the accompanying figure captions.

### Light and electron microscopy

Retinal samples of normal cat retina (n=2) and cat retinas detached for 3 days (n=2), 7 days (n=4), and 28 days (n=3) were processed for light (LM) and electron microscopy (EM) as detailed in Fisher et al. [35]. Sampled regions were excised from central superior retina. Sections were taken on an LKB V ultramicrotome (LKB-Produktor AB, Bromma, Sweden). For LM, 1-μm-thick sections were stained with saturated aqueous paraphenylenediamine and photographed using an Olympus BX60 Research microscope equipped with a Microfire digitial camera (Optronics, Goleta, CA). For EM, serial sections were collected and mounted on formvar-coated slot grids, stained with uranyl acetate followed by lead citrate, and examined at 80 kV on a JEOL JEM-1230 transmission electron microscope (JEOL USA Inc., Peabody, MA) equipped with an AMT digital camera system (Advanced Microscopy Techniques Corp., Danvers, MA). More than 40 rod spherules were examined using serial thin sections.

### Image processing

All transmitted and confocal images were captured digitally and required no additional image processing once correct exposures or laser settings were determined. Transmission EM images were taken using standard EM sheet film negatives that were scanned on a Microtek ScanMaker *i*900 (Microtek Lab, Inc., Carson, CA). The images were then inverted, the contrast was adjusted, the images assembled, and labels were applied using Adobe Photoshop CS2 software (Adobe Systems, Inc., San Jose, CA).

## Results

### Light microscopy

The most relevant morphological effects of detachment in the first month were evident in the outer retina (compare Figure 1A to Figure 1B,C; also see [13]). As photoreceptors lose their outer segments and inner segments and some rods die by apoptosis [36], rod terminals retracted from the OPL toward their cell bodies. In the OPL of the normal cat retina, rod terminals were clustered tightly together just beneath the inner edge of the ONL and above and around the cone terminals (Figure 1D). The retraction of rod spherules was underway by day 3 when there was a loosening of their clustering and evidence of a few terminals moving up into the lower ONL. The rod terminals themselves had yet to shrink by day 3 (compare Figure 1D,E). Horizontal cell hypertrophy was already evident 3 days after detachment with swollen perinuclear cytoplasm and obvious vacuoles in the OPL (Figure 1E). Rod spherule retraction was common at 7 days when they became thin, acquired a distinctive teardrop shape, and stained dark (arrows, Figure 1B,F). The retraction of so many rod terminals at this time point interrupted the tight configuration of the OPL neuropil, making it difficult to recognize in some regions (Figure 1B,F). However, these distinctive retracting terminals so obvious at 7 days were hard to find at 28 days when remaining rod nuclei fronted directly on a narrowed OPL neuropil containing swollen HC processes (Figure 1G).

**Figure 1:**
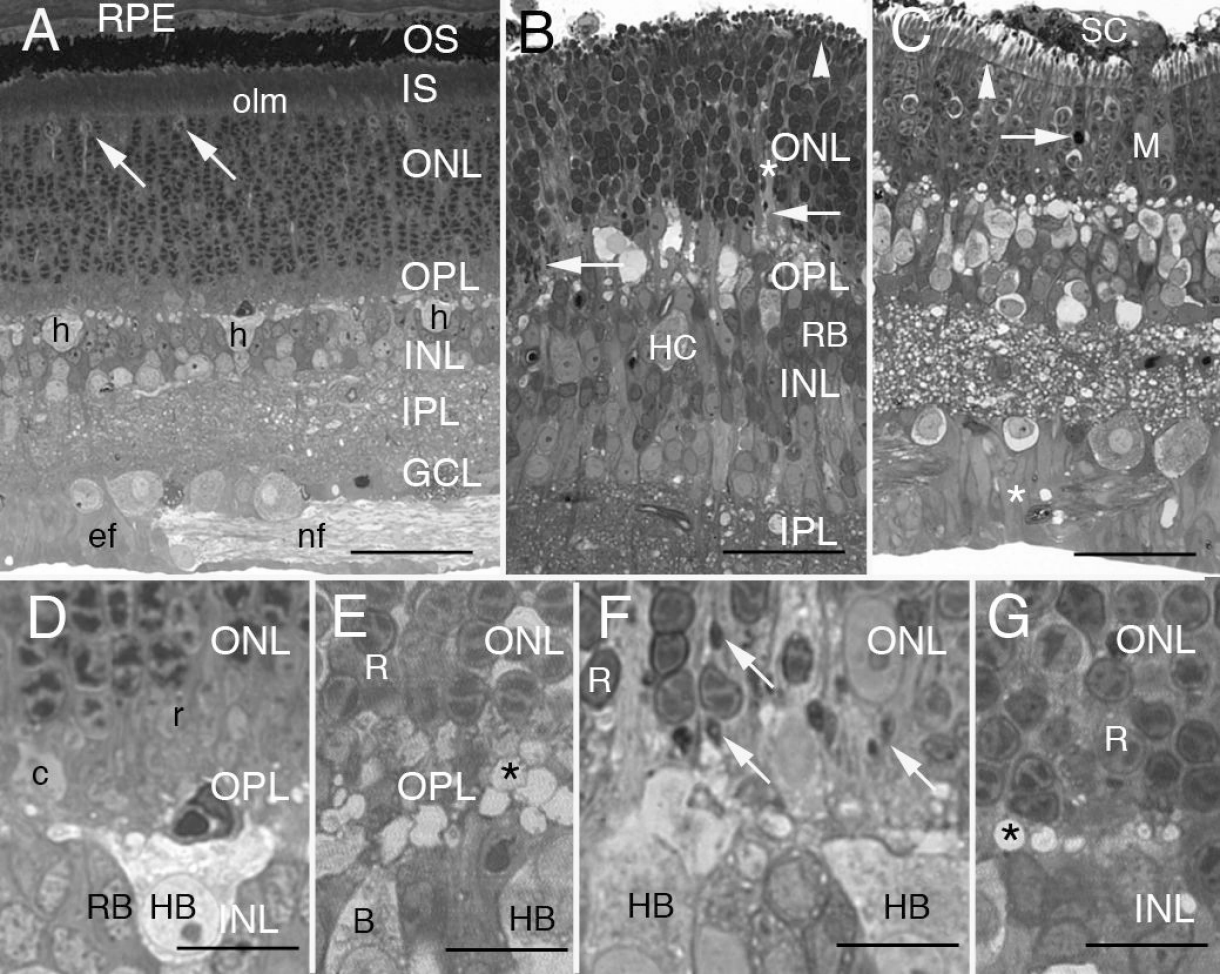
Light micrographs of 0.5 μm-thick resin sections of normal and detached cat retina stained with paraphenylenediamine. **A:** The layers of this radial section of the posterior, superior temporal region of normal cat retina are labeled to the right. Lipophilic paraphenylenediamine (PPDA) intensely stains the photoreceptor outer segments (OS). Except for the few lightly staining cone nuclei (arrows) just beneath the outer limiting membrane (olm), the 11 or more ranks of nuclei comprising the outer nuclear layer (ONL) are all rods. The outer plexiform layer (OPL) begins where the innermost row of rod nuclei ends and spans to the line of lightly stained horizontal cell somata, 3 of which are shown (h). More proximal still are the inner nuclear (INL), inner plexiform (IPL), and ganglion cell (GCL) layers. Bundles of ganglion cell axons (nf) lie among Müller cell end feet (ef), bordering the retina’s inner limiting membrane. An enlargement of the central region is shown in **D**. Abbreviations: retinal pigment epithelium (RPE); inner segments (IS). Scale bar represents 40 μm. **B:** In this slightly oblique section through the posterior, superior nasal region of 7-day detached retina, photoreceptor OS and IS have collapsed and extend just a few micrometers above the olm (white arrowhead). Only 8 to 10 rows of rod nuclei form the ONL, including regions of loosely packed cells (*). The inner margin of this layer is no longer well defined. Many densely stained retracting rod spherules lie in this region (arrows). Most of the lightly stained profiles of varying sizes in the outer OPL are the swollen processes of the B-type horizontal cell (HC). A B-type HC cell body (HB) with its prominent nucleolus lies in the outermost layer of the INL while several rod bipolar cells (RB) cluster to the right. Scale bar represents 35 μm. **C:** After 28 days of detachment, the ONL of posterior superior temporal retina has only 5 to 7 rows of rod nuclei including one cell shown here undergoing apoptosis (arrow). “Vacuoles” in the OPL are actually swollen processes of the B-type HC. Profiles of retracting spherules, so prominent at 7 days of detachment (**B**, **F**), are no longer obvious at 28 days. The Müller cells show dramatic changes including enlargement of the overlapping end feet (*), migration of their nuclei through the outer retina (M), and participation in the formation of a subretinal scar (SC) that spreads out over the photoreceptor layer. Arrowhead indicates olm. Scale bar represents 35 μm. **D:** The stratified OPL of normal cat retina is shown at higher magnification. This same region is seen in the middle of **A**. From the top of the figure, the innermost 5 to 6 rows of rod nuclei are densely packed. The outer half of the OPL contains the photoreceptor terminals: several rows of tightly packed rod spherules (r) lie distal to cone pedicles (c). The inner half of the OPL is the neuropil itself. A small capillary containing a red blood cell is transected just above the cell body of a B-type HC (HB) at the outer margin of the INL. RB nuclei also lie in the outer half of the INL. Scale bar represents 10 μm. **E:** Three days after detachment, the feline outer retina retains much of its typical stratification. Although voids appear among them, rod cell bodies (R) are still closely packed above clustered rod spherules that appear to stain less intensely than normal, but retain their normal size. The underlying neuropil is heavily populated with vacuole-like profiles (*) identified as the swollen branches of hypertrophied HC dendrites and axon telodendria. A portion of an HB shows lightly stained cortical cytoplasm ballooning past the typically dense field of organelles lying near the nucleus. B is an unidentified cone bipolar cell. Scale bar represents 10 μm. **F:** Disrupted organization of the inner ONL and OPL a week after detachment. The inner ONL has loosely packed rod nuclei (R) separated by thickened Müller cell processes. Large, lightly-staining, ectopic Muller cell nuclei are found here as are the small dark profiles of retracting rod spherules (arrows). Lightly stained HCs have swollen cell bodies (HB). The largely empty cortical cytoplasm extends beyond a diverse field of perinuclear organelles. Pale profiles scattered throughout the OPL are swollen HC processes. Scale bar represents 10 μm. **G:** Almost a month after detachment, the cell bodies of surviving rods (R) in the ONL directly front on a narrowed OPL neuropil that contains vacuole-like cross-sections of hypertrophied HC processes (*). Though not evident at this magnification, scattered spherules can still be identified by electron microscopy (Figure 4I). Scale bar represents 10 μm.

### Immunocytochemistry

As rod terminals retracted after detachment, immunocytochemical labeling revealed distinctive neurite outgrowths from both HCs and RBs. Based on labeling patterns, it has been established that most HC outgrowths (Figure 2D) arise from the axon terminal system of B-type cells [21]. Similar outgrowths from RBs (Figure 2B) were easily identified because of their specificity for labeling with anti-protein kinase C-α [14]. Neurites of both types often terminated near retracted rod terminals (arrows, Figure 2B,D). It is likely that there was both outgrowth and pruning of dendrites from RBs. In normal cat retina (Figure 2A), fine RB dendrites contacted 15–50 rod spherules [5] see Figure 1A in [14]. After detachment, these numerous fine dendrites rapidly decreased in number so that by 7 days (Figure 2B) only a few stout dendrites arose from each RB cell body. There appeared to be a similar process of simultaneous neurite outgrowth and pruning in HC axon terminals (compare Figure 2C,D). These morphological changes after detachment all suggested significant changes at the ultrastructural level in rod synapses.

**Figure 2:**
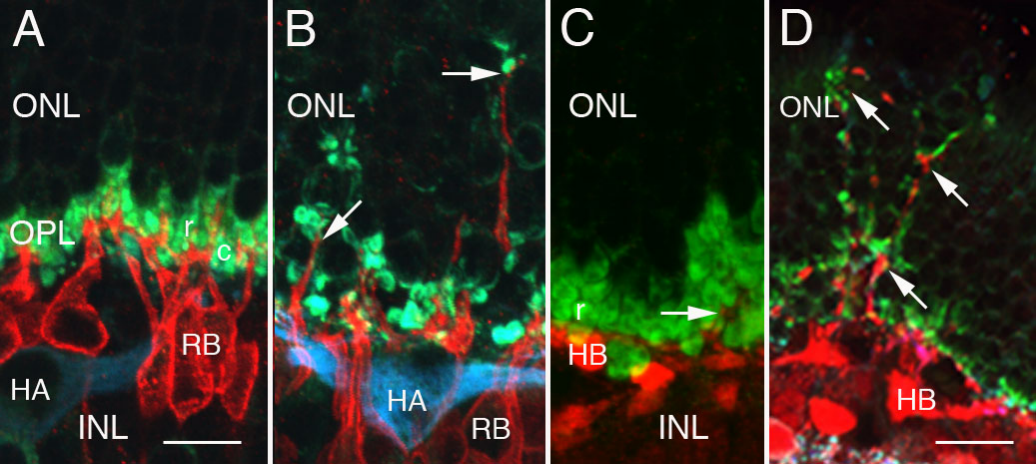
Confocal immunofluorescent images of normal and detached cat retina. **A:** This projection of a z-series consisting of 3 0.5 µm-thick optical sections of normal cat retina triple-labeled with anti-protein kinase C (PKC), anti-VAMP2, and anti-neurofilament shows the tightly-clustered synaptic terminals of rods (r) and cones (c; green). Anti-PKC (red) labels the rod bipolar (RB) cells whose apical processes cross the outer plexiform layer (OPL) neuropil to reach the rod spherules but never normally extend into the outer nuclear layer (ONL). Anti-neurofilament (blue) labels an A-type HC (HA) lying in the inner nuclear layer (INL). Scale bar represents 12 μm. **B:** This projection of a z-series consists of 5 0.5 µm-thick optical sections of retina that was detached for 28 days, sectioned, and then triple-labeled with anti-PKC, anti-VAMP2, and anti-neurofilament. Labeled RB outgrowths (red) extend  into the ONL where they terminate (arrows) adjacent to VAMP2-positive retracted rod terminals (green). The RB nuclei lie slightly deeper in the outer INL than an HA labeled with anti-neurofilament (blue). Scale bar as in **A**. **C:** This projection of a z-series consists of 3 0.5 µm-thick optical sections of normal cat retina that was double-labeled with anti-calretinin and anti-VAMP2. Rod spherules (r) labeled with anti-VAMP2 (green) are closely clumped together at the inner margin of the ONL. The cell body of a B-type HC (HB) is labeled with anti-calretinin (red). A small branch of a HC axon terminal rises to innervate several spherules (arrow) but does not extend into the ONL itself. Scale bar as in **A. D:** This projection of a z-series of 5 1.0 µm-thick optical sections of 7-day detached cat retina shows a loose assembly of anti-VAMP2-labeled rod spherules (green) in the OPL as well as retracting spherules at various depths of the ONL. Outgrowths from B-type HCs labeled with anti-calretinin (red) extend into the ONL to terminate near retracted rod spherules (arrows). The soma of a B-type HC is labeled (HB). Scale bar indicates 10 μm.

### Electron microscopy: Overall changes in or near the OPL

In normal cat retina the photoreceptor terminals and their postsynaptic processes are tightly packed together. The spaces between them are occupied by thin Müller cell processes. Typically, rod spherules (rs; Figure 3A) are arrayed together in the outer OPL between the lowest tier of rod cell nuclei and the OPL neuropil. Cone pedicles (cp; Figure 3A) tend to lie just at the outer edge of the neuropil.

**Figure 3:**
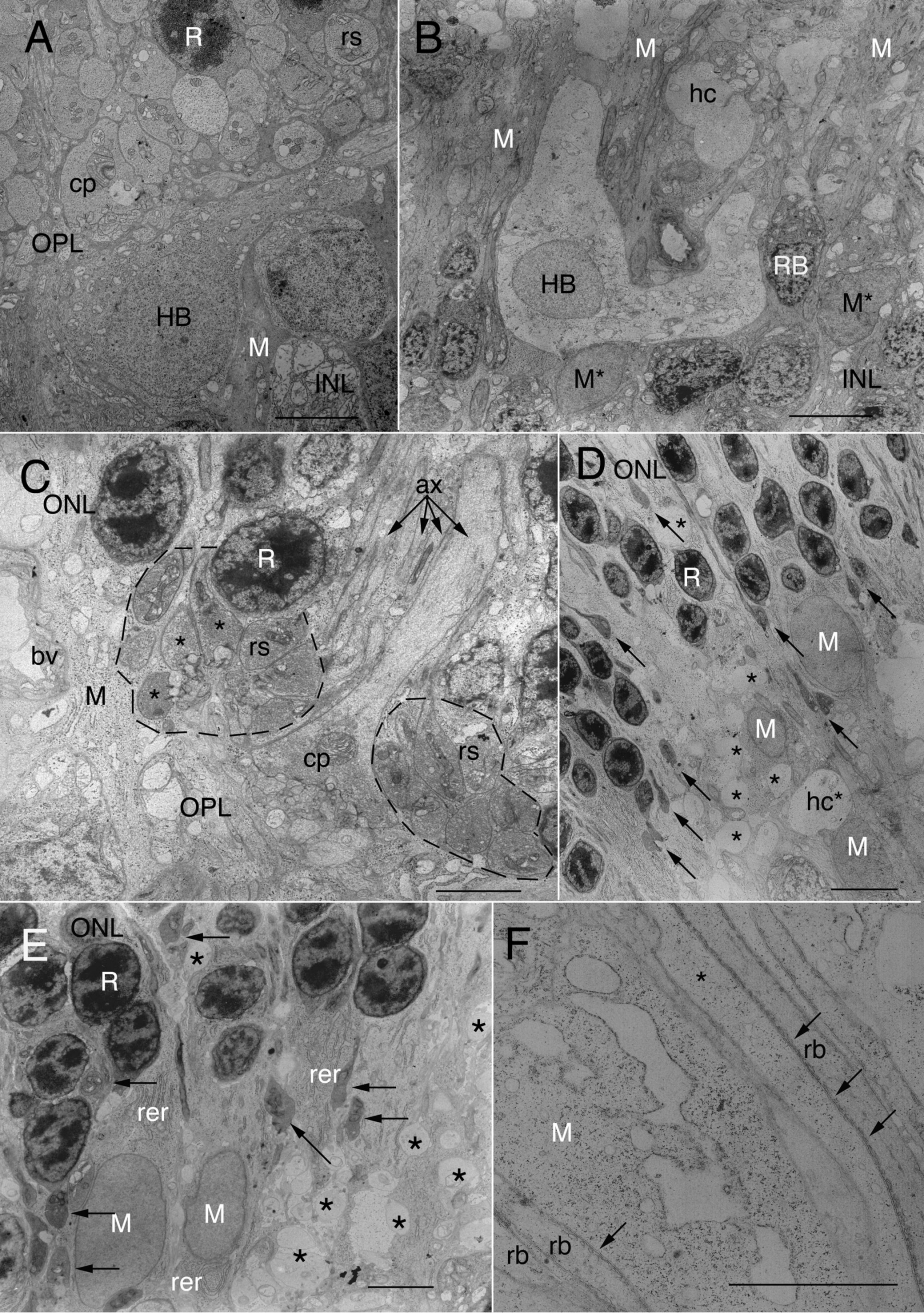
Electron micrographs of normal and detached cat retina. **A:** The cell body of a B-type horizontal cell (HB) lies at the inner edge of the outer plexiform layer (OPL). Its apical cytoplasm is densely packed with organelles. One of its main dendritic trunks courses off to the right. Many rod spherules (rs) are packed together in the outer OPL beneath the lowest tier of rod cell nuclei (R). Part of a cone pedicle is shown (cp). Dark Müller cell cytoplasm (M), though surrounding all retinal neurons, is most evident in the inner nuclear layer (INL), where Müller cell processes are thickest. Notice that Müller cell processes in the outer retina are so fine as to be unresolved between neuronal processes. Scale bar represents 10 μm. **B:** In cat retina detached for 7 days, HBs appear enlarged, resulting in much lighter staining of their cytoplasm and a decreased density of organelles. Similar lightly-stained and enlarged profiles (hc) occur throughout the OPL. These correspond to the ‘vacuoles’ in the OPL of Figure 1B,C,E-G. The OPL is disrupted lacking the typical layering of photoreceptor terminals. Müller cell cytoplasm (M) is atypically obvious in the outer OPL. Müller cell nuclei in the INL (M*) are less electron dense than usual and have rounded up, losing their typical angularity. Electron dense RB somata lie to either side of the HC, one of which is labeled. Scale bar represents 10 μm. **C:** In 7-day detached cat retina, 2 clusters (dotted outlines) of rod spherules (rs) are in their usual position above the OPL neuropil, surrounding a cone pedicle (cp). Those at the left have turned their basal synaptic surfaces toward one another; 3 of them have no hilus but have apparent postsynaptic contacts in non-invaginating or “open” configurations. (See also Figure 4C.) On the far left, a thick column of Müller cell cytoplasm has replaced lost photoreceptor terminals. The axons of rods and cones (ax) are indicated by arrows. Scale bar indicates 5 μm. **D:** Ectopic Müller cell nuclei (M) lie in the proximal ONL and OPL of 7-day detached retina. Their processes are clearly evident in the ONL where they surround surviving rod nuclei (R). Swollen HC processes (*) and dendrites (hc*) are evident deep in the ONL as well as the OPL. Electron-dense, teardrop-shaped retracting spherules (arrows) and their axons lie at varying levels in the proximal ONL. The OPL is largely filled with HC and Müller cell processes. Scale bar represents 10 μm. **E:** The outer OPL in a 7-day detached retina contains darkly-stained, elongated rod spherules (arrows) that are seen at the OPL and also within the proximal ONL amid rod nuclei (R). Large Müller cell nuclei (M) lie within the OPL in columns of cytoplasm that often contain extensive arrays of rough endoplasmic reticulum (rer), scattered mitochondria, polysomes, and cytoskeletal elements. Asterisks indicate swollen HC processes. Scale bar represents 5 μm. **F:** In 7-day detached retina, thickened rod bipolar cell dendrites (rb) and a cone axon (*) co-fasciculate through the inner ONL with a stout Müller cell process containing distended rER and many ribosomes. Subsurface cross-sections of smooth endoplasmic reticulum (arrowheads) are part of the “helical organelle” characteristic of rod bipolar cells and key to their identification by electron microscopy. Scale bar represents 5 μm.

The OPL of a cat retina detached for 7 days (Figure 3B) can be highly disorganized compared to that in normal attached cat retina (Figure 3A). No photoreceptor terminals were evident along its distal border in this region (top 1/3 of Figure 3B). However, other regions of the OPL in detached retina at both 7 and 28 days still had clusters of rod spherules in their usual position in the distal OPL. In some cases these surrounded surviving cone pedicles (Figure 3C). Particularly conspicuous at 7 days after detachment were the many electron-dense rod axons and spherules that appeared to be retracting into the ONL (arrows, Figure 3D,E), corresponding to the dark-stained structures observed by LM (Figure 1B,F). These teardrop-shaped terminals were more elongated and thinner (approximately 1–2.5 μm in width) than those in normal retina (3–5 μm) [15]. As spherules withdrew from the OPL, they lost connections with cone terminal telodendria that normally contacted a subpopulation of spherules [15]. The cone pedicles themselves underwent significant morphological changes but remained anchored in position along the border of the OPL [37,38].

After detachment, HCs appeared to undergo a type of hypertrophy, resulting in swollen perinuclear cytoplasm, enlarged dendrites and axon telodendria (Figure 3B,D,E). The perinuclear cytoplasm of the B-type HC is normally crowded with organelles [4,15,17], but these are dramatically reduced by 7 days following detachment (Figure 1F). One HC, measuring 17×14.5 μm had an 8 μm-diameter nucleus lying in a largely empty perinuclear cytoplasm (data not shown). Similar cells were seen on LM to the right in Figure 1F and by EM in Figure 3B, where two enlarged dendritic trunks from one HC course off at right angles to each other.

### The rod spherule complex: Presynaptic changes

Many detailed ultrastructural studies on normal cat retina have demonstrated that every spherule contains 1 or 2 synaptic ribbons, each associated with a “synaptic unit” comprised of 2 invaginating HC axon terminal (HCat) processes and 1 or more RB dendrites [1,4,6,15]. These innervating processes enter the base of the spherule via a single “opening” or “hilus” only 0.24 μm in diameter, through which they all must pass [1,2,6,15,39,40] (see paired arrowheads in Figure 4A,B,D). Beneath the synaptic ribbon was an electron-dense arciform density whose extent was more easily appreciated in radial aspect when the arched ribbon was viewed en face (asterisks, Figure 4B).

**Figure 4:**
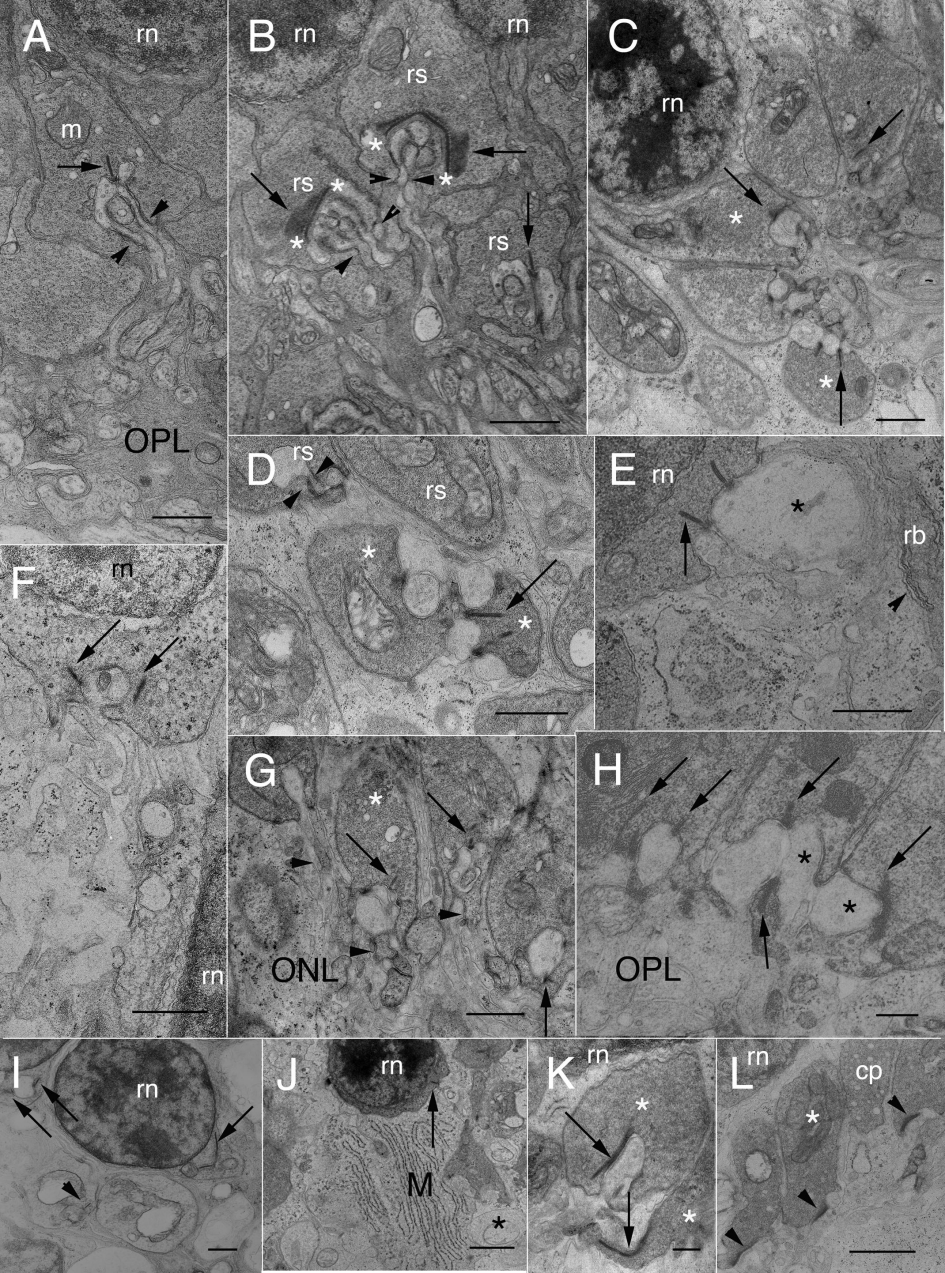
Electron micrographs of normal and detached cat retina. **A:** A rod spherule lies between the OPL neuropil  and the innermost tier of rod cell nuclei (rn). The presynaptic cytoplasm is crowded with a uniform population of 35–45 μm light cored synaptic vesicles. A synaptic ribbon (arrows) opposes fine, invaginating HCat telodendria and rod bipolar dendrites. The innervating processes pass through the hilus (arrowheads) to enter the invagination. Mitochondria (m) typically lie near the synapse. Scale bar represents 0.8 μm. **B:** In normal cat retina, 3 neighboring rod spherules (rs) show details of presynaptic and postsynaptic architecture. In two rod spherules, the synaptic ribbon (arrows) is seen chiefly en face. In addition to the dense presynaptic populations of synaptic vesicles, the upper spherule also contains profiles of ser cisternae or endosomes, two of which lie near a mitochondrion. At the base of the synaptic ribbons, asterisks (*) demarcate the ends of the particularly electron-dense arciform densities. HCat telodendria and rod bipolar (RB) dendrites pass through a basal hilus to enter their respective synaptic invaginations (arrowheads). The rod nucleus is labeled (rn). Scale bar represents 0.8 μm. **C:** This area of the OPL in 7 day detached cat retina is enlarged from Figure 3C. Although these spherules lie in their usual location and show little evidence of retraction, their ultrastructure is abnormal. The spherules themselves are turned in such a way that their basal synaptic surfaces, instead of facing the OPL, face each other around a cluster of postsynaptic processes. Two of the spherules (*) have shallow invaginations without evidence of any hilus. Swollen HCat telodendria contact these spherules in “open” configurations. Synaptic ribbons (arrows) are seen in three spherules. All the spherules in this location are filled with synaptic vesicles. Interestingly, the two profiles of a synaptic ribbon in the bottom spherule have arciform densities that appear to project as synaptic ridges between apposing horizontal cell (HC) lateral elements. Scale bar represents 0.75 μm. **D:** Several retracting rod spherules in 7-day detached cat retina lie in the lower outer nuclear layer (ONL) enveloped by Müller cell cytoplasm. The uppermost spherule is invaginated; one HC lobe is visible as is the hilus (arrowheads) through which the postsynaptic processes pass. Two subjacent spherules (*) make open contacts with swollen HC axon telodendria. One contains a synaptic ribbon (arrow) that shares a HC lobe with a neighboring ribbon. Both ribbons appear to extend from arciform densities that project outward as synaptic ridges. Spherules above and below them lack synaptic ribbons in this plane of section, but have multiple mitochondria. Synaptic vesicles populate all of the terminals. Scale bar represents 0.8 μm. **E:** A section through basal perinuclear cytoplasm of a rod soma in the mid-ONL of 7-day detached cat retina shows numerous synaptic vesicles and two cross-sections of synaptic ribbons. One to the left is presynaptic to two vesicle-containing HC processes, one of which (*) is very swollen and also postsynaptic to the other ribbon, both in open, non-invaginated configurations. Subjacent Müller cell cytoplasm has distended rer cisternae and numerous polysomes. Scale bar represents 0.8 μm. **F:** Two rod nuclei (rn) lie in the mid- to lower ONL of cat retina detached for 7 days. The shallow perinuclear synaptic invagination of the upper cell body contains two pale lateral elements and a small, dark central element. Arrows point to cross-sections of synaptic ribbons (perhaps the same one) that lack arciform densities and have only a few synaptic vesicles lying near them. Presynaptic cytoplasm also contains a few endosomes and scattered polysomes. Scale bar represents 0.6 μm. **G:** Four partially retracted spherules lie in the inner ONL of a 7-day detached retina. All have their synaptic surfaces facing the inner retina. These terminals are filled with synaptic vesicles and have synaptic ribbons (arrows) presynaptic to electron-lucent HCat telodendria. One of the spherules (*) makes an open contact with postsynaptic processes and lacks the normal synaptic invagination. RB outgrowths (arrowheads) lie near these synapses but do not enter the invaginations. Scale bar represents 0.8 μm. **H**: Three rod spherules in 7-day detached cat retina remain at the OPL but nevertheless lack typical synaptic invaginations. Presynaptic cytoplasm is crowded with synaptic vesicles. Synaptic ribbons (arrows) appose swollen and electron-lucent HCat telodendria in open configurations. The HCat processes innervating two adjacent spherules (*) directly appose each other. Scale bar represents 0.3 μm. **I:** Two rod nuclei (rn) in 28-day detached cat retina front on the OPL. Both contain a perinuclear basal invagination with synaptic ribbons (arrows). Subjacent spherules lie in their normal position; one also contains a synaptic ribbon (arrowhead). The density of the synaptic vesicle population is abnormally low. Scale bar represents 0.6 μm. **J:** Low power survey of the inner ONL in cat retina detached for 7 days. Several retracting rod spherules are loosely packed along with a rod soma (rn) containing a cross-section of an indistinct synaptic ribbon (arrow) that apposes an HCat process without evidence of any invagination, arciform density, or RB dendritic contact. Only two or three synaptic vesicles associate with the ribbon in this plane of section. Müller cell cytoplasm (M) enshrouding these structures contains prominent arrays of parallel rer cisternae. Asterisk (*) indicates swollen HCat process. Scale bar represents 1.5 μm. **K:** Two retracting spherules (*) in cat retina detached for 7 days contain synaptic ribbons (arrows) that lie almost parallel to their presynaptic membranes. Arciform densities are not discernible. The upper spherule is invaginated, the lower is not. Both are crowded with synaptic vesicles. Post-synaptic HC lateral elements contain flattened light cored vesicles of various sizes. Scale bar represents 0.3 μm. **L:** In 7-day detached cat retina, three retracting rod spherules in the lower ONL are surrounded by Müller cell cytoplasm (M). All three show basal densifications (arrowheads) but no ribbons or other structural characteristics of rod synapses. The presynaptic cytoplasm contains vesicles, one or more mitochondria (*), endosomes, and scattered polysomes. The edge of a cone pedicle (cp) is seen. Scale bar represents 1.2 μm.

Retracting terminals still possessed several of the typical components of presynaptic machinery including mitochondria, synaptic ribbons, arciform densities, and synaptic vesicles. Cat spherules usually have more than one mitochondrion that can lie close to the synaptic site, but also can extend distally into the rod axons (Figure 4L  and Figure 5A,B). Retracting terminals also contained 1 or 2 ribbons. These ribbons have a normal thickness (30–35 nm) and height (0.2–0.25 μm), but are shorter than the 2.3±0.3 μm reported for ribbons in normal cat spherules [1]. Overall, the rod spherule synaptic ribbons showed greater abnormalities in substructure with increasing displacement from the OPL, but they were never found floating free in the cytoplasm away from the membrane. The fate of the arciform density in retracting terminals was unclear since they underlay some ribbons (Figure 4C-E,H,K) but were not evident with many others (Figure 4F,J and Figure 5C). Curiously, basal membrane densifications were sometimes seen unaccompanied by any other typical presynaptic structures (Figure 4L). Normal spherules were filled with synaptic vesicles, both surrounding the synaptic ribbons and scattered throughout the presynaptic cytoplasm (Figure 4A,B). The vesicles were spherical and a uniform 35–50 nm in diameter in control as well as detached retinas (Figure 4C-H). Partially retracted terminals showed a range of vesicle populations from dense (Figure 4C,D) to sparse (Figure 4L). Fully retracted terminals, common in the 28-day detachments (Figure 4I), usually had many presynaptic features lying in their perinuclear cytoplasm, but relatively few vesicles (Figure 4F,I,J), although some had many (Figure 4E). In contrast to the 7-day detached retina, however, profiles of actively retracting terminals were rare in the 28-day detached retinas. Additionally, many rod nuclei in the outer ONL of the detached retinas apparently had no axon and lacked any of the typical presynaptic structures.

**Figure 5:**
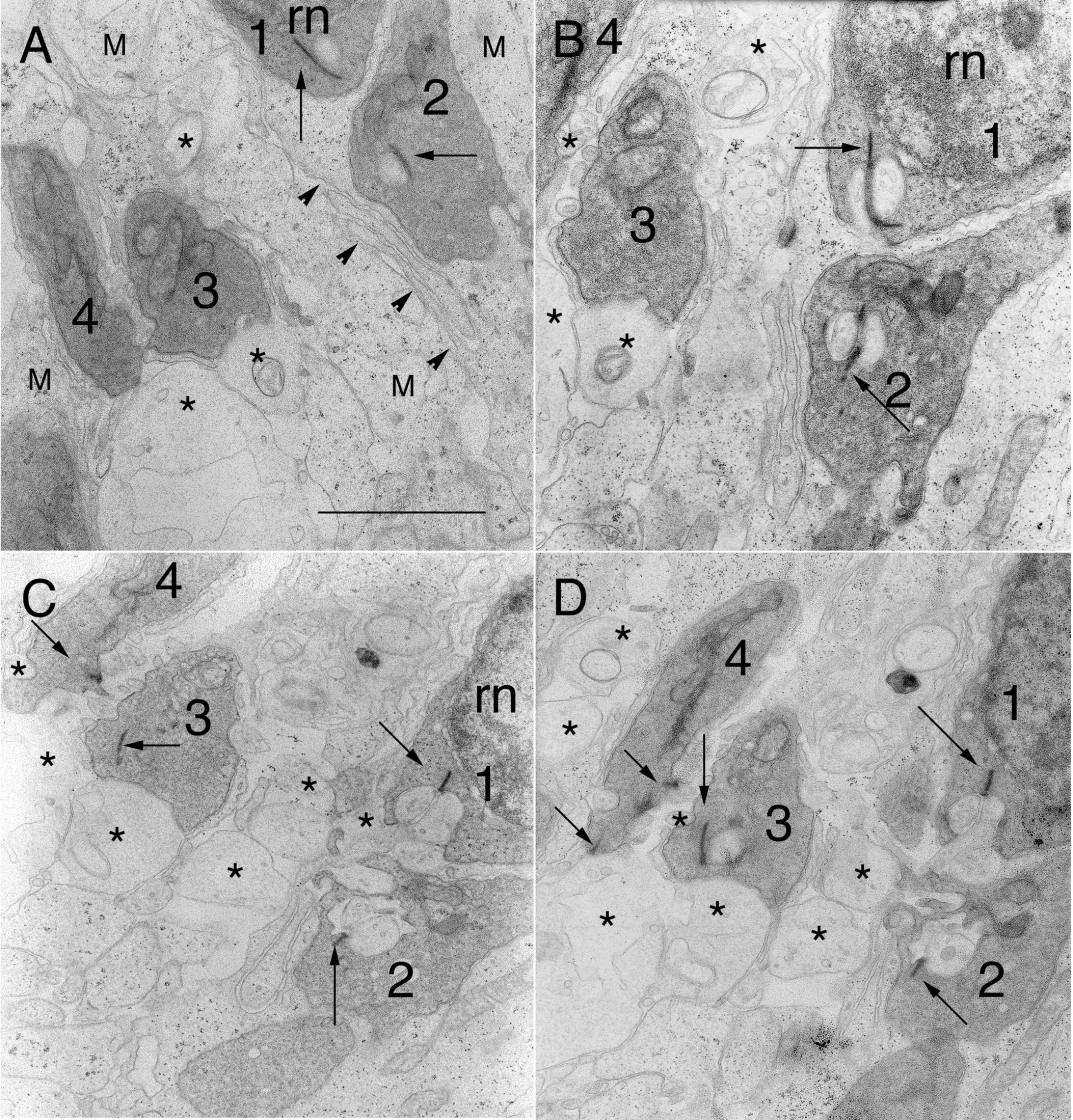
Serial electron micrographs through 4 retracted spherules in 7-day detached retina. Scale bar (in **A**) for **A-D** represents 2 μm. **A:** Spherules 1 and 2 contain synaptic ribbons (arrows), each apposing the top of their respective synaptic invaginations. Only mitochondria are seen in spherules 3 and 4. Arrowheads line the interfaces of apposing Müller cells. Swollen HCat processes (*) that lack polysomes can be differentiated from Müller cell processes (M) that have many. **B:** In the next section, the opposing lobes at each synaptic ribbon in spherules 1 and 2 are visible. They contain vesicles as do the swollen HCat processes (*) from which they arise. The hilus of spherule 1 can be seen. Another HCat process directly apposes spherule 3. **C:** Two sections later the openings to the synaptic invaginations of spherules 1 and 2 are obvious. The hilus of spherule 2 actually faces the outer nuclear layer instead of the outer plexiform layer. The horizontal cell (HC) lateral elements innervating spherules 1 and 2 connect with numerous swollen HCat processes (*) running along apposing Müller cell surfaces. Synaptic ribbons (arrows) now can be seen in spherules 3 and 4, both of which directly appose HCat processes. **D:** Two sections later, one lobe of the synaptic invagination can be seen in spherule 3 while spherule 4 makes open contact with its innervating HCat processes (*). The innervation of spherule 1 by HC lateral elements is still evident, while that of spherule 2 is not. There is no clear evidence that any of these 4 spherules have contact with rod bipolar dendrites.

### The rod spherule complex: Postsynaptic changes

After detachment (Figure 4C-L and Figure 5), the pattern of innervation of the spherules was altered. Even when spherules were in their usual location (Figure 4C), they were often turned relative to one another. The invaginations were reduced in depth, complexity, and integrity. Invagination width and depth are features that vary little from species to species. Rao-Mirotznik and coworkers [1] reported a consistent invagination depth and width of about 1 μm. Some retracting spherules in 7 day detachments had similar dimensions, especially those that have retracted the least. Overall, most had depths (and widths) ranging from 0.5 to 0.75 μm (see examples in Figure 4C,D,F-I and Figure 5C), yet many had no invaginations at all (Figure 4D,E,J). The less complex synaptic invaginations of retracting spherules in the 7 day detachments contained fewer postsynaptic processes than usual—often limited to a pair of HC lobes and no central elements from the RB dendrites (Figure 4D,E and Figure 5C). Most rod ribbons still opposed two HC processes, but many appeared to be lobes from branches of the same, rather than different, HC axon terminals as occurred in normal cat retina [4]. We also noted a lack of “fingers” of rod terminal cytoplasm projecting into the invaginations as has been reported in normal cat and human rods [1,6,39,40]. The overall integrity of the spherule invagination was altered because invariably rod terminal cytoplasm fully encapsulated the postsynaptic processes and isolated them from other spherules as well as from the fine glial processes of the encompassing Müller cells. Normally the only opening to this invagination is at its base via the narrow “hilus” [39]. Although many retracting terminals maintained a hilus, not all were at the base and, more importantly, the opening itself was frequently wider (Figure 4F,G and Figure 5C,D) or lacking altogether. In such cases HC lateral elements contacted the spherules directly in open configurations where they also opposed other swollen HCat telodendria and Müller cell processes (see * in Figure 4C-E,H and Figure 5C,D). Often the rod terminal cytoplasm encapsulating the invagination pulled back asymmetrically such that one HC lateral element was invaginated while its partner is not (Figure 4D,H; spherule 3 in Figure 5D). These open configurations can result in the swollen HC lateral elements of adjacent spherules directly apposing one another.

The dilation of the hilus after detachment and the resulting opening up of the invagination revealed another feature of rod spherule architecture: the synaptic ridge. Literally a shallow evagination that projects as a ridge between the HC lateral elements, the synaptic ridge underlay the arciform density [11] and was flanked by the 2 linear active zones along the ribbon base and immediately above the bifurcation ridge [6], where released glutamate diffused between apposed HC lobes with their AMPA receptors and spread down to the RB dendrites with their mGluR6 receptors. The trough-like arciform density has long been proposed to offer some sort of rigidity to the ribbon complex. This synaptic ridge was revealed in this study when the synaptic invagination opened and the rod terminal membrane pulled back and flattened (Figure 4C-E,H and Figure 5C,D). It projected a short distance between apposing HC lobes. This configuration may be short-lived during the retraction phase since fully retracted terminals can contain ribbons that directly appose postsynaptic processes without arciform densities (Figure 4J).

The density of cytoplasmic organelles appeared sparse in the B-type HC after detachment by comparison to that in normal feline retina where their distinctively dark axon terminal processes occupied much of the distal  OPL neuropil and contained numerous microtubules, neurofilaments, and scattered mitochondria in a granular cytoplasm [15]. Such processes were not evident in the detached cat retina, but instead the outer OPL contained a population of circular processes (Figure 1B,C,E-G) containing minimal ground substance or cytoskeleton (Figure 3B,D,E), occasional mitochondria with few cristae (Figure 5A,B), and, notably, no ribosomes (Figure 5A-D). Axon terminal processes can be differentiated from Müller cell processes because the former lack ribosomes, while the latter have many [15]. These profiles, typically ranging from 1 to 2 μm in diameter (Figure 3D,E and Figure 5A-D), often were adjacent to one another in the outer OPL, no longer separated from each other by intervening Müller cell processes. Serial sections through a small cluster of retracted and partially retracted rod spherules demonstrated that processes arising from them became the lateral elements postsynaptic to the rod ribbons (Figure 5A-D). Thus the axon terminal underwent similar ultrastructural changes to those observed in its cell body. In normal cat retina, the HC axon terminal branches inside the invagination contained a population of light-cored vesicles [15]. Similar vesicles occurred in the swollen terminal branches after detachment (Figure 4C-E,H and Figure 5B-D); they were of less uniform size than the synaptic vesicles of the rod spherules, ranging from 45 to 110 nm in diameter with most of the smaller vesicles being circular and the larger ones more elongated. The swollen processes contacting the spherules varied in size from 0.4 up to 0.7 μm in diameter, much larger than typical HC lateral elements (approximately 0.25 μm in diameter) [15]. The processes of the OPL neuropil in detached retinas were surrounded by expanded extracellular space, a feature never associated with the tightly packed OPL of normal retina.

The RB dendritic trunks that grew into the ONL after detachment (Figure 3F and Figure 4E) were quite stout, 0.3 to 0.5 μm in caliber. Key to their identification was the presence of the “helical organelle,” a system of spiraling and flattened tubules of smooth endoplasmic reticulum that immediately underlay the cell’s membrane [39,40]. Small, dark, vermiform terminal processes only 0.08-μm thick abruptly branched off from these RB dendritic trunks. These branchlets lacked organelles and coursed along Müller cell processes until they contacted their target spherules. Most, however, ultimately terminated close to a spherule but did not enter its invagination (Figure 4C,G).

### Müller cell changes

In response to retinal detachment, Müller cell nuclei were seen in the distal retina (Figure 3D,E). At 7 days after detachment, the nuclei of migrating Müller cells were ovoid, averaging 7×5 μm with faint nucleoplasm devoid of heterochromatin (Figure 1F and Figure 3B,D,E) and differed in appearance from normal Müller cell nuclei, which were compact, angular, electron-dense and restricted to the inner portions of the inner nuclear layer (INL). While Müller cell processes in the normal ONL were thin and inconspicuous, after detachment their hypertrophy created dense radial columns of Müller cell cytoplasm full of intermediate filaments, microtubules, ribosomes, scattered mitochondria, and large arrays of rough endoplasmic reticulum (Figure 3B,C,E,F and Figure 4E,J). As the packing density of cells in the ONL declined and rod terminals retracted from the OPL, remaining retinal processes appeared juxtaposed to, rather than ensheathed by, these thickened Müller cell processes (Figure 3D,E, Figure 4D-F,J,L, and Figure 5A).

## Discussion

The first few days after detachment represent a critical period of outer segment degeneration, RPE apical surface remodeling, the beginning of Müller cell reactivity, and a wave of apoptotic cell death that can kill up to 20% of the rod population [13,36]. After this, apoptosis proceeds but subsides, Müller cell hypertrophy continues until large glial scars form intra- and extraretinally. Seemingly of critical importance to information processing by the retina, photoreceptor synaptic terminals undergo a series of changes resulting in significant alteration to this critical primary synapse. By day 3 of this critical period, many rod terminals have begun the process of retraction to their respective cell bodies. The end product is a large population of rods with their terminals far distal to the OPL and showing a highly modified structural arrangement. Their deep synaptic invaginations are lost and synaptic ribbons are shortened with, in some cases, a greatly reduced population of vesicles. By a month after detachment fewer terminals appear in the active state of retraction as if a new steady-state has been attained [38]. Rod terminal retraction has been described in many retinal degenerations as well as in sheets of porcine photoreceptors prepared for retinal transplantation [31], suggesting that it might be a common response to photoreceptor injury. The underlying mechanisms leading to retraction are yet to be fully understood, although recent studies have suggested that the RhoA-ROCK pathway as well as cAMP may be involved [32,41].

Terminal retraction is accompanied by dramatic changes in second order neurons, easily identified by specific confocal immunolabeling studies in several species and several retinal degenerations. The combination of rod terminal retraction and neurite sprouting by the postsynaptic targets, the RBs and B-type HCats [14,21], suggests that rod synaptic transmission has been compromised.

The rod spherule in the feline retina has been well studied and its characteristics measured in detail [1,2,6]. The release of 100 vesicles/second/spherule is estimated to be the minimum rate of glutamate exocytosis necessary to account for the rod’s broad range of sensitivity [2,42]. Unique among CNS neurons, the rod photoreceptor relies on a single active zone to transmit its signal. The synaptic zone is folded, not planar as in most other CNS synapses, and its cleft volume is 20 times larger than that of most conventional synapses [for review see 10]. This synaptic interface is contained within a single deep invagination of the rod axon terminal. The volume of the extracellular space within the invagination averages 0.21 μm^3^ and varies little from spherule to spherule, and the length of the linear active zone is remarkably similar across species at approximately 2.5 μm [1,6]. This allows glutamate concentrations to be achieved within the cleft sufficient to simultaneously stimulate glutamate receptors on the invaginating processes of both RBs and HCats [1,2]. It is thought that glutamate receptors inside the synaptic invagination as well as those clustered outside prevent “spillover” of glutamate from one spherule to another that could degrade signal fidelity [see 2,11]. While the exact mechanism by which synaptic ribbons regulate tonic glutamate release remains controversial [10], the ribbon’s surface area is essentially invariant [1]. A ribbon will typically have around 130 docking sites for vesicles primed for release (twice the number found at the largest conventional synapses known), along with 5 times that number of vesicles tethered to the ribbon above the linear active zone. This type of data points to some central role of the ribbon’s architecture in achieving the rate of transmitter exoctyosis necessary for rod function. Our results showed that much of the critically important organization of the rod spherule is drastically altered as spherules retract following retinal detachment.

It is well documented that photoreceptor outer segments undergo severe degeneration within a week of detachment. Our data showed that rod spherules also undergo structural “degeneration” that seems likely to affect the physiology of synaptic transmission. Like the outer segment, many rod synapses “deconstruct” their highly structured three-dimensional organization. Significantly, the elaborate fingers of rod cytoplasm that project into the invagination and may effect local glutamate concentration [1,2] appear highly vulnerable to the effects of injury. We found little evidence of such fingers in the retracting terminals. The opening up or virtual loss of the rod hilus also reflects an important step in the restructuring of the invagination. In general, the amount of structural disruption to a spherule’s invagination correlates with the degree of its displacement from the OPL, but even then the severity of this deconstruction can vary between spherules—some have invaginations of almost normal depth (spherule 1, Figure 5), others are shallower (spherule 2, Figure 5), some have partial invaginations where only one lobe of a pair of HCat lateral elements is surrounded by rod cytoplasm (Figure 4G,H, spherule 3, and Figure 5), and others have no invagination whatsoever (* in Figure 4D,E). In the latter case, it is particularly striking to see images of paired HCat lobes lined up on either side of a ribbon, both in direct contact with the spherule but also lying exposed to other neuronal and glial cell processes in the altered OPL neuropil. Regulating cleft volume and preventing glutamate spillover would seem impossible in these highly altered synapses.

Another serious challenge to normal spherule function following detachment is the loss of synaptic contacts with other cells— particularly with RBs. Fewer than half of the spherules we examined in serial sections had any recognizable RB contacts. That RBs also experience dendritic pruning, leaving only 1 to 4 neurite outgrowths per cell recognizable by immunofluorescence microscopy [14,21], would also suggest that many spherules lose their RB innervation, thus compromising the rod pathway. Dendritic pruning of RBs has also been reported in other retinal degenerations [43-46]. There is no specific evidence that any of the contacts made by the modified spherules are functional. While spherules in normal cat retina receive contact from two different RBs [5], we never saw evidence of more than one RB dendrite ending close enough to a retracted spherule to warrant classifying it as a “contact.” These RB dendritic outgrowths probably retained their mGluR6 receptors, because they were found on ectopic RB dendritic outgrowths in the *nob2* mutant mouse [23,24], in the bassoon knockout mouse [26], and in the RCS rat [22], so that if contact was reestablished after retinal detachment normal synaptic transmission could resume. Even though our study revealed that processes from HCats were much more successful in remaining closely apposed with retracting spherules, rarely did we see spherules contacted by more than one axon terminal system, a distinct difference from the situation in normal cat retina [4]. In general, there were fewer postsynaptic HC processes in the remaining invaginations, and they appeared undifferentiated and structurally abnormal.

Another consequence of retinal detachment in feline retina is the extreme hypertrophy that the B-type HCs and Müller cells undergo. The swollen, pale B-type HC cell bodies and dendritic trunks in detached samples were immediately apparent by both LM and EM. Our data indicate that this swelling also affects the terminals that contact rod spherules. Thus B-type HCs are highly reactive to detachment with abnormal swelling, robust neurite sprouting, and an upregulation of neurofilaments [21,47,48]. HC hypertrophy is reminiscent of reactions seen in axotomized neurons elsewhere in the CNS where, despite an increase in neurofibrillar components, the cells otherwise appear swollen and sparse in organelles. These HCs however are not axotomized by detachment but may experience a reduction in synaptic input to their axon terminals as rods undergo degeneration.

The more familiar example of cell hypertrophy after retinal detachment is that seen in the early reaction of Müller cells [13,49,50]. According to our data, Müller cell processes fill in areas of the OPL where rod spherules and other neuronal processes are lost. HC and Müller cell hypertrophy results in a rapid change in the immediate cellular environment for the surviving rod terminals and cone terminals. Large expanses of Müller cell cytoplasm now populate the OPL. Although there is no physiologic data, the extensive hypertrophy of the B-type cells may well alter their electrical properties. Thus retinal detachment leads to many changes in the photoreceptor synaptic milieu; as photoreceptors degenerate, terminals and postsynaptic processes are lost, and there is a distinct loosening of the densely packed OPL. Processes generated by the hypertrophy of Müller cells can result in large columns of fibrillar Müller cell cytoplasm interrupting the regular array of neurons in the ONL as well as processes in the OPL.

Limiting the loss of photoreceptor outer segments has always been thought to be a key element in lessening damage caused by retinal detachment. However, preventing the remodeling of photoreceptor synaptic terminals and subsequent loss of synaptic connectivity may be just as important to visual recovery. This study represents a start at understanding the structural reactions of photoreceptor synapses to detachment. There is still much to be learned. Serial section studies of this kind are time-consuming, tedious, and limited in resolution in the z axis. A promising approach may be to study photoreceptor synapses in relatively thick sections by electron tomography with intermediate voltage EM [51]. Similar studies of ribbon synapses in the ear have uncovered striking levels of detail not available in studies of the kind done here [52].

## References

[1] Rao-Mirotznik R, Harkins AB, Buchsbaum G, Sterling P (1995). Mammalian rod terminal: architecture of a binary synapse.. Neuron.

[2] Rao-Mirotznik R, Buchsbaum G, Sterling P (1998). Transmitter concentration at a three-dimensional synapse.. J Neurophysiol.

[3] Steinberg RH, Reid M, Lacy PL (1973). The distribution of rods and cones in the retina of the cat (Felis domesticus).. J Comp Neurol.

[4] Kolb H (1974). The connections between horizontal cells and photoreceptors in the retina of the cat: electron microscopy of Golgi preparations.. J Comp Neurol.

[5] Boycott BB, Kolb H (1973). The connexions between bipolar cells and photoreceptors in the retina of the domestic cat.. J Comp Neurol.

[6] Migdale K, Herr S, Klug K, Ahmad K, Linberg K, Sterling P, Schein S (2003). Two ribbon synaptic units in rod photoreceptors of macaque, human, and cat.. J Comp Neurol.

[7] Dowling JE. The Retina: An Approachable Part of the Brain. Cambridge: Harvard University Press; 1987.

[8] Burns ME, Augustine GJ (1995). Synaptic structure and function: dynamic organization yields architectural precision.. Cell.

[9] von Gersdorff H, Vardi E, Matthews G, Sterling P (1996). Evidence that vesicles on the synaptic ribbon of retinal bipolar neurons can be rapidly released.. Neuron.

[10] Sterling P, Matthews G (2005). Structure and function of ribbon synapses.. Trends Neurosci.

[11] Heidelberger R, Thoreson WB, Witkovsky P (2005). Synaptic transmission at retinal ribbon synapses.. Prog Retin Eye Res.

[12] Rodieck RW. The First Steps in Seeing. Sunderland, MA: Sinauer Associates, Inc; 1998.

[13] Erickson PA, Fisher SK, Anderson DH, Stern WH, Borgula GA (1983). Retinal detachment in the cat: the outer nuclear and outer plexiform layers.. Invest Ophthalmol Vis Sci.

[14] Lewis GP, Linberg KA, Fisher SK (1998). Neurite outgrowth from bipolar and horizontal cells after experimental retinal detachment.. Invest Ophthalmol Vis Sci.

[15] Kolb H (1977). The organization of the outer plexiform layer in the retina of the cat: electron microscope observations.. J Neurocytol.

[16] Kolb H, Nelson R (1983). Rod pathways in the retina of the cat.. Vision Res.

[17] Fisher SK, Boycott BB (1974). Synaptic connexions made by horizontal cells within the outer plexiform layer of the retina of the cat and the rabbit.. Proc R Soc Lond B Biol Sci.

[18] Sterling P (1983). Microcircuitry of the cat retina.. Annu Rev Neurosci.

[19] Kolb H, Nelson R (1984). Neural architecture of the cat retina.. Prog Retin Eye Res.

[20] Sterling P, Freed MA, Smith RG (1988). Architecture of rod and cone circuits to the *On*-beta ganglion cell.. J Neurosci.

[21] Linberg KA, Lewis GP, Matsumoto B, Fisher SK (2006). Immunocytochemical evidence that rod-connected horizontal cell axon terminals remodel in response to experimental retinal detachment in the cat.. Mol Vis.

[22] Cuenca N, Pinilla I, Sauvé Y, Lund R (2005). Early changes in synaptic connectivity following progressive photoreceptor degeneration in RCS rats.. Eur J Neurosci.

[23] Chang B, Heckenlively JR, Bayley P, Brecha NC, Davisson MT, Hawes NL, Hirano AA, Hurd RE, Ikeda A, Johnson BA, McCall MA, Morgans CW, Nusinowitz S, Peachey NS, Rice DS, Vessey KA, Gregg RG (2006). The *nob2* mouse, a null mutation in *Cacna1f*: anatomical and functional abnormalities in the outer retina and their consequences on ganglion cell visual responses.. Vis Neurosci.

[24] Bayley PR, Morgans CW (2007). Rod bipolar cells and horizontal cells form displaced synaptic contacts with rods in the outer nuclear layer of the *nob2* retina.. J Comp Neurol.

[25] tom Dieck S, Altrock WD, Kessels MM, Qualmann B, Regus H, Brauner D, Fejtová A, Bracko O, Gundelfinger ED, Brandstätter JH (2005). Molecular dissection of the photoreceptor ribbon synapse: physical interaction of bassoon and RIBEYE is essential for the assembly of the ribbon complex.. J Cell Biol.

[26] Specht D, tom Dieck S, Ammermüller J, Regus-Leidig H, Gundelfinger ED, Brandstätter JH (2007). Structural and functional remodeling in the retina of a mouse with a photoreceptor synaptopathy: plasticity in the rod and degeneration in the cone system.. Eur J Neurosci.

[27] Haeseleer F, Imanishi Y, Maeda T, Possin DE, Maeda A, Lee A, Rieke F, Palczewski K (2004). Essential role of Ca^2+^-binding protein 4, a Ca_v_1.4 channel regulator, in photoreceptor synaptic function.. Nat Neurosci.

[28] Takada Y, Vijayasarathy C, Zeng Y, Kjellstrom S, Bush RA, Sieving PA (2008). Synaptic pathology in retinoschisis knockout (Rs1-/y) mouse retina and modification by rAAV-Rs1 gene delivery.. Invest Ophthalmol Vis Sci.

[29] Claes E, Seeliger M, Michalakis S, Biel M, Humphries P, Haverkamp S (2004). Morphological characterization of the retina of the CNGA3^−/−^Rho^−/−^ mutant mouse lacking functional cones and rods.. Invest Ophthalmol Vis Sci.

[30] Wang Q-P, Jammoul F, Duboc A, Gong J, Simonutti M, Dubus E, Craft CM, Ye W, Sahel JA, Picaud S (2008). Treatment of epilepsy: the GABA-transaminase inhibitor, vigabatrin, induces neuronal plasticity in the mouse retina.. Eur J Neurosci.

[31] Khodair MA, Zarbin MA, Townes-Anderson E (2003). Synaptic plasticity in mammalian photoreceptors prepared as sheets for retinal transplantation.. Invest Ophthalmol Vis Sci.

[32] Khodair MA, Zarbin MA, Townes-Anderson E (2005). Cyclic AMP prevents retraction of axon terminals in photoreceptors prepared for transplantation: an in vitro study.. Invest Ophthalmol Vis Sci.

[33] Lewis GP, Linberg KA, Geller SF, Guérin CJ, Fisher SK (1999). Effects of the neurotrophin BDNF in an experimental model of retinal detachment.. Invest Ophthalmol Vis Sci.

[34] Hale IL, Matsumoto B. Resolution of subcellular detail in thick tissue sections: immunohistochemical preparation and fluorescence confocal microscopy. In: Matsumoto B, editors. Cell Biological Applications of Confocal Microscopy. 2nd ed. San Diego: Academic Press; 2002. p. 301–355.

[35] Fisher SK, Anderson DH, Erickson PA, Guérin CJ, Lewis GP, Linberg KA (1993). Light and electron microscopy of vertebrate photoreceptors including a technique for electron microscopic autoradiography.. Methods Neurosci.

[36] Cook B, Lewis GP, Fisher SK, Adler R (1995). Apoptotic photoreceptor degeneration in experimental retinal detachment.. Invest Ophthalmol Vis Sci.

[37] Lewis GP, Sethi CS, Linberg KA, Charteris DG, Fisher SK (2003). Experimental retinal reattachment: a new perspective.. Mol Neurobiol.

[38] Fisher SK, Lewis GP, Linberg KA, Verardo MR (2005). Cellular remodeling in mammalian retina: results from studies of experimental retinal detachment.. Prog Retin Eye Res.

[39] Missotten L. The Ultrastructure of the Human Retina. Brussels: Arscia. Uitgaven N.V.; 1965.

[40] Linberg KA, Fisher SK (1988). Ultrastructural evidence that horizontal cell axon terminals are presynaptic in the human retina.. J Comp Neurol.

[41] Fontainhas AM, Townes-Anderson E (2008). RhoA and its role in synaptic structural plasticity of isolated salamander photoreceptors.. Invest Ophthalmol Vis Sci.

[42] van Rossum MCW, Smith RG (1998). Noise removal at the rod synapse of mammalian retina.. Vis Neurosci.

[43] Strettoi E, Pignatelli V (2000). Modifications of retinal neurons in a mouse model of retinitis pigmentosa.. Proc Natl Acad Sci USA.

[44] Strettoi E, Porciatti V, Falsini B, Pignatelli V, Rossi C (2002). Morphological and functional abnormalities in the inner retina of the rd/rd mouse.. J Neurosci.

[45] Pignatelli V, Cepko CL, Strettoi E (2004). Inner retinal abnormalities in a mouse model of Leber’s congenital amaurosis.. J Comp Neurol.

[46] Cuenca N, Pinilla I, Sauvé Y, Lu B, Wang S, Lund RD (2004). Regressive and reactive changes in the connectivity patterns of rod and cone pathways of P23H transgenic rat retina.. Neuroscience.

[47] Fisher SK, Lewis GP (2003). Müller cell and neuronal remodeling in retinal detachment and reattachment and their potential consequences for visual recovery: a review and reconsideration of recent data.. Vision Res.

[48] Coblentz FE, Radeke MJ, Lewis GP, Fisher SK (2003). Evidence that ganglion cells react to retinal detachment.. Exp Eye Res.

[49] Erickson PA, Fisher SK, Guérin CJ, Anderson DH, Kaska DD (1987). Glial fibrillary acidic protein increases in Müller cells after retinal detachment.. Exp Eye Res.

[50] Lewis GP, Matsumoto B, Fisher SK (1995). Changes in the organization of cytoskeletal proteins during retinal degeneration induced by retinal detachment.. Invest Ophthalmol Vis Sci.

[51] Chen X, Winters CA, Reese TS (2008). Life inside a thin section: tomography.. J Neurosci.

[52] Lenzi D, Crum J, Ellisman MH, Roberts WM (2002). Depolarization redistributes synaptic membrane and creates a gradient of vesicles on the synaptic body at a ribbon synapse.. Neuron.

